# Intrinsic neural network dynamics underlying the ability to down-regulate emotions in male perpetrators of intimate partner violence against women

**DOI:** 10.1007/s00429-023-02696-x

**Published:** 2023-09-09

**Authors:** Sofia Amaoui, Agar Marín-Morales, Cristina Martín-Pérez, Miguel Pérez-García, Juan Verdejo-Román, Carmen Morawetz

**Affiliations:** 1 Mind, Brain and Behavior Research Center (CIMCYC), Granada, Spain; 2 Department of Personality, Evaluation & Psychological Treatment, University of Granada, Granada, Spain; 3 Department of Psychobiology, University of Valencia, Valencia, Spain; 4 Department of Psychology, Universidad de Valladolid, Campus María Zambrano. Plaza de la Universidad 1, 40005, Segovia, Spain; 5 Department of Psychology, University of Innsbruck, Innsbruck, Austria

**Keywords:** Intimate partner violence against women, Male perpetrators, Emotion regulation, Reappraisal, Resting-state fMRI, Spectral dynamic causal modelling, Effective connectivity

## Abstract

Research has pointed to difficulties in emotion regulation as a risk factor for perpetrating intimate partner violence against women (IPVAW). While efforts have been made to understand the brain mechanisms underlying emotion regulation strategies such as reappraisal, little is known about the intrinsic neural dynamics supporting this strategy in male perpetrators. Resting-state functional magnetic resonance imaging was used to characterise the network dynamics underlying reappraisal. Spectral dynamic causal modelling was performed to examine the effective connectivity (EC) within a predefined reappraisal-related brain network. 26 men convicted for an IPVAW crime [male perpetrators] were compared to 29 men convicted of other crimes [other offenders] and 29 men with no criminal records [non-offenders]. The ability to down-regulate emotions in response to IPVAW stimuli was used as a covariate to explore its association with male perpetrators' EC. The analysis revealed that (1) compared to non-offenders, both convicted groups exhibited increased EC within prefrontal areas, enhanced EC from prefrontal to temporoparietal regions and decreased EC in the opposite direction; (2) male perpetrators compared to other offenders showed increased EC from temporoparietal to prefrontal regions and, increased EC from the supplementary motor area to frontal areas; (3) connections involving dorsolateral prefrontal cortex were found to be potential predictors of the ability to down-regulate emotions. The study provides a deeper characterisation of the brain architecture of the processes that underlie IPVAW. This knowledge could inform the work of adaptive emotion regulation strategies in intervention programmes for male perpetrators in order to reduce the high recidivism rates.

**Supplementary Information:**

The online version contains supplementary material available at 10.1007/s00429-023-02696-x.

## Introduction

Intimate partner violence against women (IPVAW) is the most severe expression of inequality and power relations of men over women (World Health Organization [WHO] 2018). It includes any act of violence (physical, psychological and sexual) exercised over women by those men who are or have been linked to them by an intimate relationship. According to the last estimates, about 38% of femicides worldwide are committed by male intimate partners, and 22–31% of all women have been subjected to violence from an intimate male partner at least once in their lifetime (WHO 2018). The societal and personal consequences for survivors of IPVAW are severe. Women survivors suffer from life-long physical, sexual and (neuro)psychological sequelae (Fernández-Fillol et al. 2021; Bacchus et al. 2018; Daugherty et al. 2019). These statistics provide compelling evidence for a more detailed understanding of the neuronal mechanisms underlying IPVAW perpetration in order to predict and prevent this sort of violence (Bueso-Izquierdo et al. 2016a).

The aetiologic mechanisms and risk factors implicated in IPVAW are highly diverse (Patró-Hernández 2017). Therefore, one efficacious approach to reducing global incidence would be identifying common characteristics. Recent research points to difficulties in emotion regulation among male perpetrators of IPVAW as being one consistent risk factor for the expression of violence (Marín-Morales et al. 2021; Berke et al. 2019). Emotion regulation describes the ability to effectively manage emotional experiences by applying cognitive strategies to either up- or down-regulate their intensity (Gross 2002). Effective emotion regulation is associated with several positive outcomes, such as improved mental and physical well-being (Singh and Mishra 2011), adaptive social behaviour (Marroquín et al. 2017), and quality of our personal and professional relationships (Fischer et al. 2016). In contrast, emotion dysregulation represents a transdiagnostic factor related to a large diversity of conditions, including major depression, anxiety disorders (Gross and Jazaieri 2014), substance use disorders (Wilcox et al. 2016), schizophrenia (Horan et al. 2013), and other diagnoses (Aldao et al. 2016). Additionally, deficits in emotion regulation have been associated with lower empathy for others (Zaki 2020) and greater use of violence to solve conflicts (Roberton et al. 2014).

So far, in particular, one cognitive strategy has been studied most in violent populations, namely reappraisal (e.g., Walker et al. 2022; Barlett and Anderson 2011). Reappraisal is the reinterpretation of an emotion-eliciting situation in a way that alters its meaning and changes its emotional impact (Gross 2015). It is a key strategy of violence prevention since it requires re-evaluating the intentionality, the responsibility of the situation and the severity of the consequences (Lila et al. 2013) before the emotional response is fully aroused, which also makes it a transversal key component of IPVAW intervention programmes (Lila et al. 2013; Maloney et al. 2022). Based on the above advantages, behavioural research demonstrates that the use of reappraisal to down-regulate emotions reduces indicators of anger provocation (Mauss et al. 2007) and aggressive behaviours (Shorey et al. 2015; Barlett and Anderson 2011). These results align with studies focusing on intimate partner violence experiences, pointing out that male perpetrators struggle to regulate negative emotions, resulting in violent reactions against their partners (McNulty and Hellmuth 2008). In addition, studies showed that male perpetrators who used reappraisal to down-regulate anger provocation tended to articulate less aggressive verbalisations (Birkley and Eckhardt 2019).

At a neural level, a large body of literature examined the neural architecture underlying reappraisal (e.g., Morawetz et al. 2020; Etkin et al. 2015; Kohn et al. 2014). Reappraisal is based upon a wide-spread network including frontal and temporoparietal regions: the ventrolateral and dorsolateral prefrontal cortex (VLPFC and DLPFC) involved in cognitive control and top-down regulation (Sturm et al. 2016), the middle temporal area (MTA) suggested to play an intermediary role between prefrontal and subcortical areas (Ochsner et al. 2012), the supplementary motor area (SMA) associated to emotion processing balance between preparation and behaviour (Morawetz et al. 2016), and the temporoparietal junction/superior temporal gyrus (TPJ/STG) implicated in self-reference and the attribution of mental states and intentions to others (Schurz et al. 2017; Kohn et al. 2014). Notably, a meta-analysis has demonstrated that the large-scale network underlying reappraisal is modulated by the regulation goal (up- or down-regulation) and the stimulus valence (Sokolowski et al. 2022; Morawetz et al. 2017).

While the neural basis of reappraisal in healthy individuals is well understood, literature investigating the neural networks underlying emotion regulation difficulties in male perpetrators is sparse (Marín-Morales et al. 2021). To the best of our knowledge, to date, only one functional magnetic resonance imaging (fMRI) study examined the neural underpinnings of reappraisal in this specific group of men (Marín-Morales et al. 2021). The study showed that during the down-regulation condition, male perpetrators reported increased activation of the right VLPFC when viewing IPVAW pictures compared to other offenders. They also exhibited greater activation of the right ventral anterior cingulate cortex and left insula while viewing IPVAW stimuli versus negative stimuli during the down-regulation condition. In addition, a recent resting-state fMRI study (Amaoui et al. 2022) investigated the functional connectivity in male perpetrators compared to two control groups (non-offenders and other offenders) and reported increased functional connectivity between reappraisal core brain regions, specifically, between MTA, DLPFC and VLPFC and between DLPFC and SMA. These findings suggest that male perpetrators show a specific brain functioning associated with reappraisal strategy.

However, it remains unknown how the ability to down-regulate emotions is related to intrinsic brain connectivity. Moreover, functional connectivity analyses are correlational; thus, no causal inferences could be drawn about the neural architecture underlying reappraisal in male perpetrators. Using spectral dynamic causal modelling (spDCM; Friston et al. 2014) it is possible to examine brain network dynamics without a task, thereby revealing information about baseline connectivity patterns. This novel technique allows the estimation of effective connectivity parameters such as the connection strength, the directionality and whether a specific connection is more likely to exert an excitatory or an inhibitory effect on another region (Stephan et al. 2010). This neural information could be related to behavioural and cognitive manifestations of emotion regulation difficulties and, in turn, partially explain violent behaviour. Given the recent development of neuroscience in the field of IPVAW, it could be of particular relevance to use this intriguing possibility of spDCM analysis to identify possible biomarkers that could be used in intervention studies.

The present preregistered study (https://osf.io/auj2m) investigates for the first time the effective (directed causal) connectivity at rest within the brain network support reappraisal to down-regulate emotions (obtained from a recent meta-analysis; Morawetz et al. 2022) in men convicted for an IPVAW crime and relates it to the ability to down-regulate emotions. Given the preciousness of a dataset of men convicted for an IPVAW crime, this work represents an extension of the previously published studies (Marín-Morales et al. 2021; Amaoui et al. 2022). In the present research, we aimed to determine whether the task effect (i.e. emotional state ratings obtained during task-based fMRI; Marín-Morales et al. 2021) is—to some extent—related to the effective connectivity of the reappraisal network at rest. To address this issue, spDCM represents the optimal analysis approach. Given the sparse literature on resting-state brain connectivity of male perpetrators (Amaoui et al. 2022), we hypothesised that: (1) male perpetrators would demonstrate different patterns of effective connectivity within the reappraisal brain network compared to both control groups, and (2) that the intrinsic effective connectivity of male perpetrators will be modulated by the ability to down-regulate negative emotions when viewing IPVAW-related stimuli.

## Methods

### Participants

**Fig. 1:**
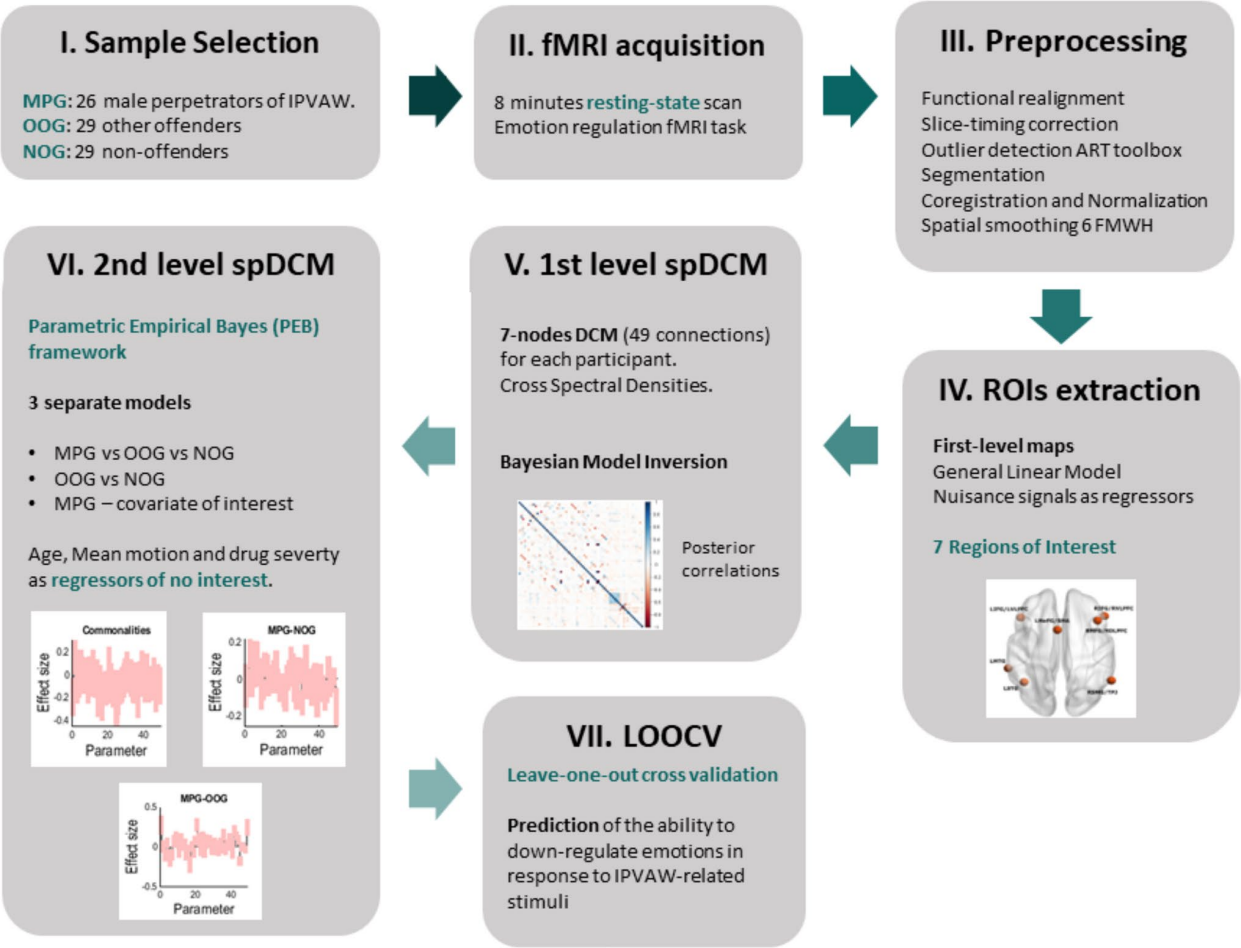
Pipeline of the procedure steps. Brief recap of the key steps for spectral dynamic causal modelling (spDCM) analysis

A total of 84 men aged 18 years or older participated in the present study: 26 male perpetrators (MPG; mean age = 41.19, SD = 9.71), 29 other offenders (OOG; mean age = 38.97, SD = 11.05) and 29 non-offenders (NOG; mean age = 38.28, SD = 8.54). Inclusion criteria were defined as follows. The MPG (male perpetrator group) consisted of men convicted of an intimate partner violence crime against women, which, according to Spanish law "*covers any act of physical, psychological and sexual violence exercised over women by those who are or have been their male spouses or by those who are or have been linked to them by a similar relationship*" (Law 1/2004, Comprehensive Protection Law against Intimate Partner Violence, IPV). The OOG (other offender group) consisted of men convicted of a crime unrelated to IPVAW, such as drug trafficking, dangerous driving or scams (no crime against persons was included). The NOG (non-offender group) consisted of men with no previous criminal records. All groups shared the following exclusion criteria: neurological disease, antecedents of drug or alcohol dependence (based on the Diagnostic and Statistical Manual of Mental Disorders 4th ed; DSM-IV), illiteracy and the presence of contraindications for the MRI scanning. In addition, participants from the NOG and OOG that obtained a score equal to or greater than 11 on the severity scale of the Conflict Tactic Scale-2 (Loinaz et al. 2012) were excluded. This criterion was added to ensure that none of the participants from both control groups had a previous history of IPVAW. Although we are aware of the multiple expressions/manifestations of the violence against women, we select the most common score used in literature (Bueso-Izquierdo et al. 2016b; Verdejo-Román et al. 2019; Marín-Morales et al. 2021; 2022a,b). A detailed description of the sample is shown in Table 1 and the full procedure is illustrated in Fig. 1.

**Table 1:** Sociodemographic background, crime characteristics and emotion regulation self-reports of the 3 groups

Variables	MPG (n = 26)	OOG (n = 29)	NOG (n = 29)	`F/ χ^2^	*p*-value
*Sociodemographic background*					
Age (years)	41.19 (9.71)	39.00 (11.05)	38.28 (8.54)	0.66	.51
Years of education	9.19 (4.30)	9.55 (3.58)	9.86 (2.44)	0.25	.77
Drug severity	1.11 (.40)	1.09 (.36)	0.91 (.33)	2.61	.08
*Loss consciousness*					
Yes (< 30 min)	3.8% (1)	3.4% (1)	0% (0)	2.66	.95
Yes (< 15 min)	19.3% (5)	13.7% (4)	20.6% (6)		
No	77% (20)	82.7% (24)	79.3% (23)		
*Crime characterisation*					
CTS-2					
Severity of violence	4.27 (6.27)	0.24 (.51)	0.31 (.93)	11.43	< .00
Psychological aggression	3.62 (2.28)	1.55 (1.62)	1.31 (1.49)	13.19	< .00
Physical aggression	2.04 (1.99)	0.24 (.51)	0.31 (.93)	17.33	< .00
Sexual coercion	0.65 (1.19)	0.07 (.26)	0.14 (.36)	5.41	< .00
Type of crime					
	PV = 57.7% (15)	SCF = 10.3% (3)			
	PPV = 42.3% (11)	DD = 17.24% (5)			
		GAR = 24.1% (7)			
		DT = 34.5% (10)			
		AA = 3.4% (1)			
		UM = 10.3% (3)			
Self-report emotion regulation measures*					
DERS					
Non acceptance of negative emotional responses	12.88 (6.54)	12.61 (5.92)	11.64 (5.18)	.33	.71
Difficulties engaging in goal-directed behaviour	11.76 (4.25)	11.07 (4.35)	11.31 (3.84)	.19	.83
Difficulties controlling impulsive behaviour	11.62 (5.84)	11.72 (5.75)	10.07 (3.23)	.89	.41
Lack of emotional awareness	15.00 (4.63)	12.75 (4.48)	14.79 (3.42)	2.41	.09
Limited access to ER strategies	14.04 (6.48)	13.04 (5.98)	12.52 (5.57)	.41	.67
Lack of emotional clarity	9.08 (2.84)	8.41 (3.49)	8.36 (2.68)	.46	.63
ERQ					
Cognitive reappraisal	30.79 (7.11)	29.59 (6.86)	29.34 (7.46)	.30	.74
Expressive suppression	18.20 (6.58)	16.00 (6.45)	15.28 (5.09)	1.67	.19
*Behavioural variable emotion regulation*					
Difficulty to down-regulate	.72 (.76)	.60 (.95)	.42 (.84)	.87	.42
fMRI Mean movement	.29 (.13)	.21 (.08)	.22 (.08)	4.61	.01

MPG = male perpetrator group; OOG = other offender group; NOG = non-offender group; CTS-2 = Conflict Tactic Scale-2; drug severity variable (log10 normalised); PV = psychological violence; PPV = physical and psychological violence; SCF = scams or crimes of forgery; DD = dangerous driving; GAR = grave assault/robbery; DT = drug trafficking; AA = attack on authority; UM = unspecified misdemeanour (lost answers). DERS = Difficulties in Emotion Regulation Scale; ERQ = Emotion Regulation Questionnaire. * Due to technical problems, the data of 1–2 participants were lost, see Supplemental File for more details

This study is part of a larger project approved by the Research Ethics Committee of the University of Granada in Spain (number issued: 1000/CEIH/2019). The participants belonging to the convicted groups were collected from the Social Integration Centre "Matilde Cantos Fernández" in Granada (Spain). Non-offenders were recruited through academies and social media. Afterwards, they were requested to fill out the informed consent form voluntarily and anonymously (Organic Law 3/2018, December 5). All participants received 50 euros for participating in the study, but no penal benefit was given to the convicted groups.

### Questionnaires/self-report data

#### Sociodemographic background

The interview evaluating the *Risk of Serious Couple Violence* (Echeburúa et al. 2008) was self-administered under the supervision of a qualified research psychologist. This report assesses general sociodemographic information about both the victim and the perpetrator, the violence committed during the relationship, vulnerability factors of the victim and relationship status. Finally, information was collected regarding other crimes, drug abuse/dependence, head injury, childhood and family support.

#### Crime characterisation

The Spanish version of the *Conflict Tactic Scale-2* (Loinaz et al. 2012) was used to evaluate the severity, frequency and intensity of the violence committed during the last year before the experiment and throughout the whole relationship. It comprises two levels of severity (minor or severe) in 5 different subscales (physical violence, psychological aggression, sexual coercion, damages and negotiation). The score that will be used is calculated from the severity subscale that mainly focused on physical violence as suggested by Straus (2004).

#### Emotion regulation assessment

##### Difficulties in Emotion Regulation Scale

The *Difficulties in Emotional Regulation Scale* (DERS; Gratz and Roemer 2004) in its Spanish version (Hervás and Jódar 2008) evaluates issues in different emotional regulation aspects through 28 items assessed on a 5- point Likert scale ranging from 1 "almost never" to 5 "almost always". It is composed of the following six subscales: (a) lack of emotional awareness; (b) non-acceptance of emotional responses; (c) difficulty engaging in goal-directed behaviour; (d) impulse control difficulties; (e) limited access to emotion regulation strategies; and (f) lack of emotional clarity. In each subscale, the higher the score, the greater the difficulties in emotional regulation.

##### Emotional Regulation Questionnaire

The *Emotional Regulation Questionnaire* (ERQ; Gross and John 2003) in its Spanish version (Cabello et al. 2013). It is a 10-item scale designed to measure the tendency to regulate emotions in two ways: cognitive reappraisal (6 items) and expressive suppression (4 items). Participants answer each item on a 7-point Likert scale ranging from 1 "strongly disagree" to 7 "strongly agree". Within each subscale, the higher the score, the greater the use of the emotional regulation strategy.

### Emotion regulation task

To meet the objectives of the present study, we only used the subjective emotional state scores that participants reported during an emotion regulation fMRI task performed in the same session and following the resting-state acquisition (for more information see Supplemental Material).

### MRI data acquisition

See all the information in the Supplemental Material.

### Analyses

#### Behavioural data

Behavioural data were analysed using the Statistical Package for the Social Sciences, version 22 (SPSS; Chicago, IL, USA). ANOVAs or contingency tables (depending on the type of variable) were carried out in order to verify that there were no significant between-group differences in the sociodemographic (i.e. age, drug consumption, brain injury) and crime variables. Differences were found in drug consumption and consequently, a new variable named "drug severity" was created by summing the affirmative responses to the DSM-IV criteria for alcohol and drugs, adding the intensity and frequency of the consumption. Drug severity was used as a control variable in all fMRI analyses.

The emotional state ratings of the emotion regulation task-based fMRI (Marín-Morales et al. 2021) were used to calculate the ability to down-regulate emotions in response to IPVAW-related stimuli. This variable^1^ was calculated by subtracting the mean emotional state during the 'Decrease' condition in response to IPVAW-related stimuli from the mean emotional state during the 'Decrease' condition in response to negative IPVAW-unrelated stimuli. This means that the higher the value, the higher the difficulty to down-regulate the emotional state while viewing IPVAW-related stimuli. This variable was used as a covariate of interest in the connectivity analyses.

#### fMRI data

##### Preprocessing

Brain images were preprocessed using the Functional Connectivity CONNv20b Toolbox (Whitfield‐Gabrieli and Nieto‐Castanon 2012) running under Matlab R2020a (MathWorks, Natick, MA, USA). Preprocessing comprised: (1) functional realignment and slice timing correction; (2) outlier detection using ART toolbox; (3) extraction of potential confounding effects, including 5 principal components from cerebrospinal areas, 5 components from white matter, 12 motion regressors and regressors of noise components (one for each identified outlier scan); (4) segmentation of the structural and functional data; (5) coregistration of functional images using each participant's anatomical scans; (6) normalisation of the functional images; (7) reslice to a 2-mm voxel size in Montreal Neurological Institute space (MNI); and (8) spatial smoothing using a 6-mm FWHM isotropic Gaussian Kernel. Due to group differences in motion during the scanning, mean motion value was used as a control variable in all subsequent fMRI analyses.

##### ROI selection

**Fig. 2:**
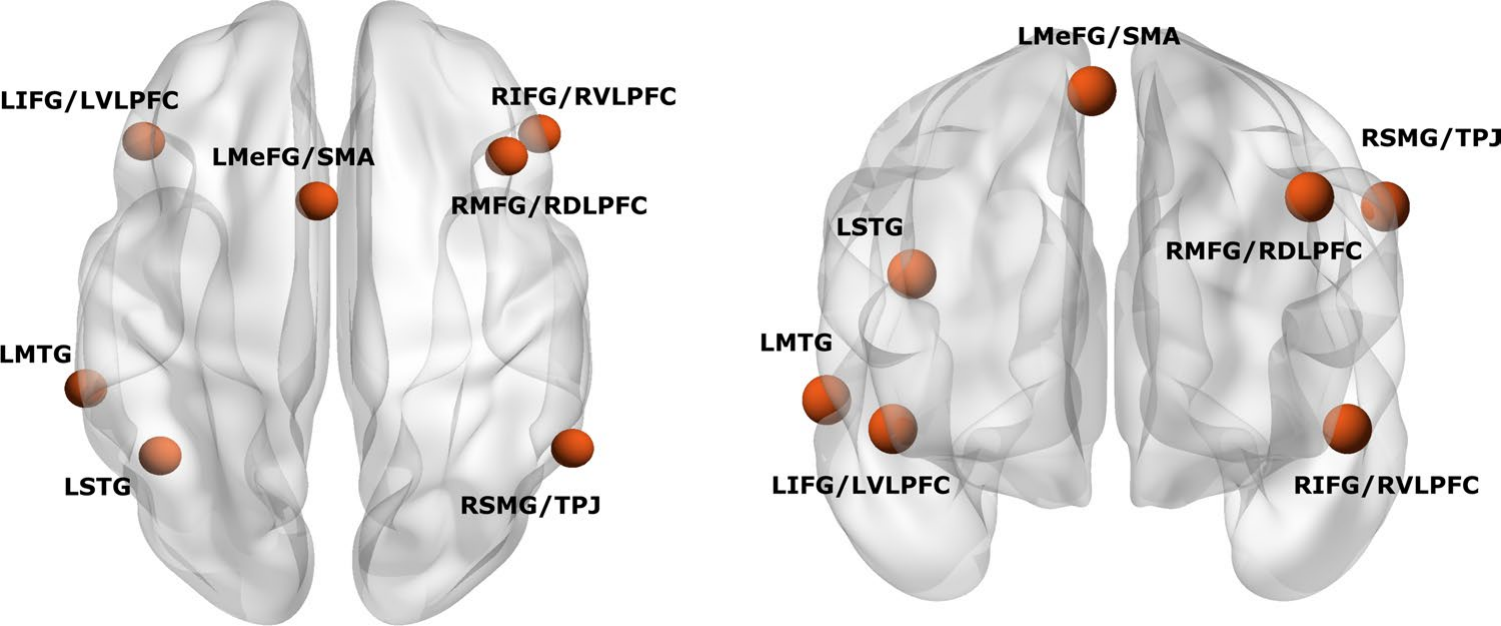
Selected anatomical region of interest (ROIs). 7 ROIs were selected from a recent meta-analysis of Morawetz et al. (2022). ROIs radius of all seeds = 6 mm. LIFG = left inferior frontal gyrus; RIFG = right inferior frontal gyrus; LMeFG = left medial frontal gyrus; RMFG = right middle frontal gyrus; LMTG = left middle temporal gyrus; LSTG = left superior temporal gyrus; RSMG = right supramarginal gyrus

Seven regions of interest (ROIs) representing the brain network supporting reappraisal to down-regulate emotions were selected from a recent meta-analysis by Morawetz et al (2022). The ROIs include: the left inferior frontal gyrus (LIFG), right inferior frontal gyrus (RIFG), right middle frontal gyrus (RMFG), left medial frontal gyrus (LMeFG), left middle temporal gyrus (LMTG), left superior temporal gyrus (LSTG) and right supramarginal gyrus (RSMG). ROIs were defined as spheres with a radius of 6 mm. The MNI coordinates are reported in Table 2 and illustrated in Fig. 2. MarsBaR Toolbox (http://marsbar.sourceforge.net) was used to extract the time courses for each ROI. First-level maps were estimated in a general linear model (GLM) in SPM12 (www.fil.ion.ucl.ac.uk/spm) by including each ROIs' time courses and nuisance signals (motion, white matter, CSF time series and invalid scans) as regressors of no interest. Finally, a high-pass filter with a 128-s cutoff period was used.

**Table 2:** Coordinates of regions of interest (ROIs)

ROIs	R/L	Shorthand term		MNI Coordinates		
				x	y	z
Inferior frontal gyrus/ ventrolateral prefrontal cortex	L	LIFG	LVLPFC	−46	28	−8
Inferior frontal gyrus/ ventrolateral prefrontal cortex	R	RIFG	RVLPFC	50	30	−8
Middle frontal gyrus/ dorsolateral prefrontal cortex	R	RMFG	RDLPFC	42	24	40
Medial frontal gyrus/ supplementary motor area	L	LMeFG	LSMA	−4	12	62
Middle temporal gyrus	L	LMTG	LMTA	−60	−38	−2
Superior temporal gyrus	L	STG	STG	−42	−56	24
Supramarginal gyrus/temporoparietal junction	R	RSMG	RTPJ	58	−54	38

##### Spectral dynamic causal modelling

This study examined resting-state effective connectivity using DCM12 implemented in SPM12. Each participant specified a fully connected 7-nodes DCM (49 connections) without exogenous inputs. Unlike stochastic DCM, Cross Spectral Densities (CSD) are used for modelling rs-fMRI data in the frequency domain instead of the time domain. This transformation allows for a more efficient inversion of the full model (Friston et al. 2014). Bayesian Model Inversion (BMI) was based on the standard variational Laplace procedure. This method uses Free Energy as a proxy for (log) model evidence (Zeidman et al. 2019a). As a result, the percentage of variance explained by the model for each participant ranged from 79.24% to 93.54%, which reflects good data fit for each estimated model. Specific diagnostics of the success of model inversion for each participant are presented in Supplemental Figures S1 and S2.

For the second-level analyses, hierarchical models over the parameters were specified within the Parametric Empirical Bayes (PEB) framework (Friston et al. 2016). Three separate Bayesian models were assessed: (1) differences in effective connectivity of MPG versus NOG and OOG; (2) differences in effective connectivity of OOG versus NOG; (3) changes in effective connectivity in male perpetrators modulated by the ability to down-regulate emotions in response to IPVAW-related stimuli. All models included age, drug severity and mean motion as regressors of no interest. For the first two models, groups were modelled as a covariate of interest. The first model included a first vector [1 (MPG), 0 (OOG), – 1 (NOG)] and a second vector [1 (MPG), – 1 (OOG), 0 (NOG)]. The second group model included a first vector [0 (MPG), 1 (OOG), -1 (NOG)] and a second vector [– 1 (MPG), 1 (OOG), 0 (NOG)]. The third model included only the male perpetrator group and the behavioural covariate of interest. The ability to down-regulate emotions in response to IPVAW-related stimuli was first mean-centred (each participant's value minus the mean group) and then included as variable of interest in the model. Bayesian Model Reduction (BMR) was applied to restrict the parameters and connectivity strengths to find the best model to explain the data. This exploratory approach assumes that all reduced models are equally probable a priori and discards those parameters that do not contribute to model evidence (Zeidman et al. 2019b) Bayesian Model Average (BMA) was calculated, and models were compared using log Bayesian model evidence. A posterior probability of 95% was used as a threshold for inference.

##### Predictive validity: cross-validation

To test if specific male perpetrators' effective connectivity could predict the ability to down-regulate emotions in response to IPVAW-related stimuli, a leave-one-out cross-validation (LOOCV) was performed (Zeidman et al. 2019b). In the analysis, a PEB model was fitted to all but one participant, and covariates for the left-out participant were predicted. The accuracy of the prediction was assessed using a threshold of ≥ 95%. A correlation between the predicted and real value of the covariate (i.e. the ability to down-regulate emotions in response to IPVAW-related stimuli) was calculated to quantify the model validation. Pearson's correlation between the connectivity strength and the covariate was calculated to estimate the effect size of the effective connectivity–behaviour association.

## Results

### Control variables for effective connectivity analyses

There were no between-group differences in any of the sociodemographic variables (age, years of education or head injury). Finally, no significant differences were found between the three groups in the self-report emotion regulation measures nor in the ability to down-regulate emotions in response to IPVAW-related stimuli tested in the task-based fMRI (Marín-Morales et al. 2021). Results are reported in detail in Table 1. Differences were found in mean motion during fMRI scanning and in drug consumption. Therefore, a variable of drug severity was created by summing the frequency and intensity of the use and the affirmative criteria of the DSM-IV for alcohol and drugs. Then, this variable was normalised and used as a confounding variable in all analyses. Age, drug severity and motion were used as control variables in the effective connectivity analyses.

### Between-group differences in effective connectivity

#### Criminal men (male perpetrators and other offenders) versus non-offenders

**Fig. 3:**
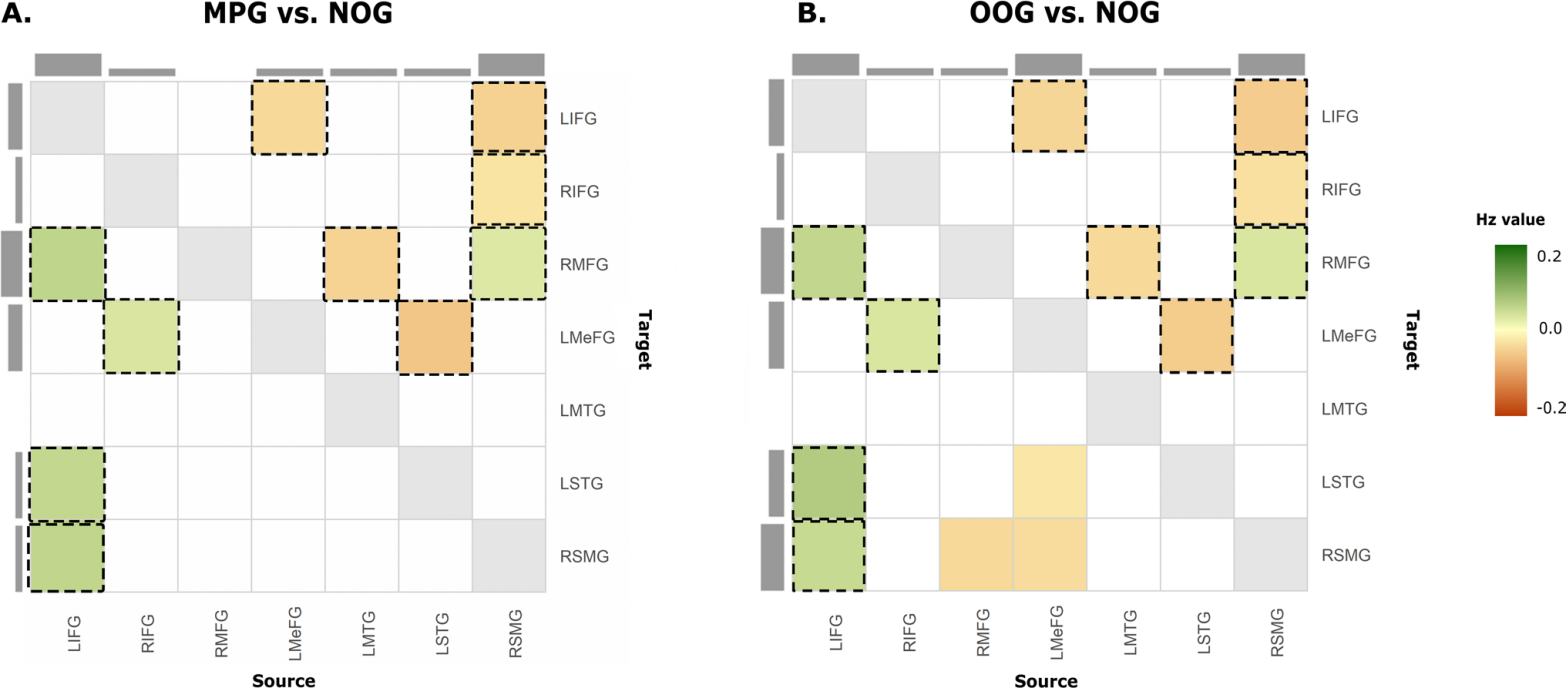
Effective connectivity differences between convicted men and non-offenders.** A** Male perpetrators (MPG) compared to non-offenders (NOG). **B** Other offenders (OOG) compared to non-offenders (NOG). Positive values (green) indicate increased connectivity for MPG and OOG compared to NOG. Negative values (orange) indicate reduced connectivity for MPG and OOG compared to NOG. Effective connectivity can be interpreted from source (column) to target (row). The connections highlighted in black are those that overlap in both comparisons. LIFG = left inferior frontal gyrus; RIFG = right inferior frontal gyrus; LMeFG = left medial frontal gyrus; RMFG = right middle frontal gyrus; LMTG = left middle temporal gyrus; LSTG = left superior temporal gyrus; RSMG = right supramarginal gyrus

*Male perpetrator group (MPG) versus non-offender group (NOG)* Half of the significant connections (5 out of 10) demonstrated increased connectivity in male perpetrators compared to non-offenders. Specifically, male perpetrators showed increased connectivity within frontal areas, from the RIFG to LMeFG and from the LIFG to RMFG. In addition, increased connectivity was observed from the frontal areas (LIFG) to temporoparietal areas (LSTG and RSMG), while reduced effective connectivity was found from the temporoparietal areas (LMTG, LSTG and RSMG) to frontal areas (bilateral IFG and LMeFG) in male perpetrators compared to non-offenders. All connections were inhibitory, except for one from the LMTG to RMFG. Of note, LIFG was found to be the brain region with the most input and output connections. Results are shown in Fig. 3a and Table 3.

**Table 3:** Significant differences in effective connectivity between male perpetrators and other offenders compared to non-offenders

Network	Connectivity			Group comparison	Effect size in Hz
	Source		Target		
MPG-NOG					
*Inhibition*					
	LIFG	→	RMFG	+	0.13
	LIFG	→	LSTG	+	0.13
	LIFG	→	RSMG	+	0.13
	RIFG	→	LMeFG	+	0.08
	RSMG	→	RMFG	+	0.07
	LMeFG	→	LIFG	−	−0.09
	LSTG	→	LMeFG	−	−0.14
	RSMG	→	LIFG	−	−0.11
	RSMG	→	RIFG	−	−0.07
*Excitation*	LMTG	→	RMFG	−	−0.11
OOG-NOG					
*Inhibition*	LIFG	→	RMFG	+	0.13
	LIFG	→	RSMG	+	0.12
	LIFG	→	LSTG	+	0.16
	RIFG	→	LMeFG	+	0.08
	RSMG	→	RMFG	+	0.08
	LMeFG	→	LIFG	−	−0.10
	LMeFG	→	LSTG	−	−0.10
	LMeFG	→	RSMG	−	−0.09
	RSMG	→	LIFG	−	−0.13
	RSMG	→	RIFG	−	−0.08
*Excitation*	LMTG	→	RMFG	−	−0.06
	LSTG	→	LMeFG	−	−0.13
	RMFG	→	RSMG	−	−0.10

MPG = male perpetrator group; OOG = other offender group; NOG = non-offender group; group comparison always in relation to the convicted groups (MPG and OOG). Increased connectivity in MPG or OOG compared to NOG is represented by " + ", while decreased connectivity in MPG or OOG compared to NOG is represented by "-". Left inferior frontal gyrus (LIFG), right inferior frontal gyrus (RIFG), right middle frontal gyrus (RMFG), left medial frontal gyrus (LMeFG), left middle temporal gyrus (LMTG), left superior temporal gyrus (LSTG) and right supramarginal gyrus (RSMG)

*Other offender group (OOG) versus non-offender group (NOG)* The analysis yielded a large amount of overlap with the previous comparison (as illustrated in Fig. 3b by black squares). Specifically, other offenders demonstrated increased effective connectivity within frontal regions (from bilateral IFG to RMFG and LMeFG) compared to non-offenders. Moreover, other offenders also showed increased connectivity from the frontal (LIFG) to temporoparietal regions (LSTG and RSMG), but decreased connectivity in the opposite direction. Finally, unlike male perpetrators, other offenders also demonstrated decreased connectivity from frontal regions (LMeFG and RMFG) to temporoparietal areas (LSTG and RSMG) compared to non-offenders. 77% of the connections (10/13) were inhibitory and 33% were excitatory. Results are presented in Fig. 3b and Table 3.

#### Between criminal groups

**Fig. 4:**
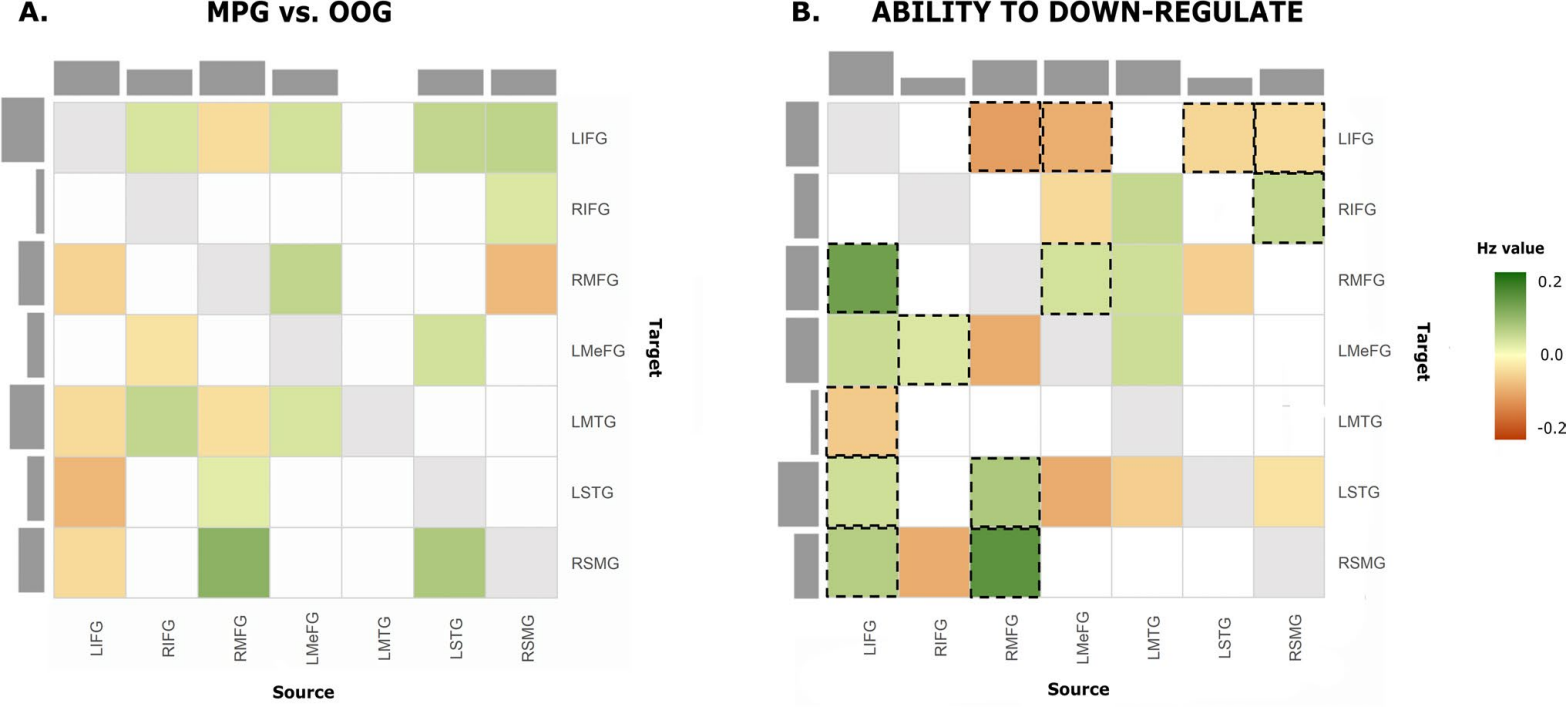
Effective connectivity (EC) differences between convicted men and association of ability to down-regulate in response to IPVAW-related stimuli with specific effective connectivity of male perpetrators.** A** Male perpetrators (MPG) compared to other offenders (OOG). **B** Association between the ability to down-regulate emotions in response to IPVAW-related stimuli and specific effective connectivity within male perpetrator group. Positive values (green) indicate increased connectivity for MPG compared to OOG and positive association between the covariate of interest and EC within male perpetrators. Negative values (orange) indicate reduced connectivity for MPG compared to OOG and negative association between the covariate of interest and EC within male perpetrators. Effective connectivity can be interpreted from source (column) to target (row). The connections highlighted in black are those that overlap in both analyses. LIFG = left inferior frontal gyrus; RIFG = right inferior frontal gyrus; LMeFG = left medial frontal gyrus; RMFG = right middle frontal gyrus; LMTG = left middle temporal gyrus; LSTG = left superior temporal gyrus; RSMG = right supramarginal gyrus

*Male perpetrator group (MPG) versus other offender group (OOG)* 60% of all connections (12/20) were increased in male perpetrators and 40% were reduced compared to other offenders. Within frontal areas, decreased connectivity was found from bilateral IFG to RMFG and LMeFG, while a reverse pattern was found. No clear pattern was found for the connections from the frontal to temporoparietal regions, however, we observed increased connectivity from the RMFG and LMeFG to temporoparietal areas, but reduced connectivity from LIFG to temporoparietal regions (LMTG, LSTG and RSMG). Finally, male perpetrators also reported increased connectivity from the temporoparietal areas (LSTG and RSMG) to frontal areas (bilateral IFG and LMeFG). Similar to the previous contrasts, more connections were inhibitory. 60% of all connections modulated by the group factor were inhibitory and 40% were excitatory. Results are shown in Fig. 4a and Table 4.

**Table 4:** Significant differences in effective connectivity between male perpetrators and other offenders

Network	Connectivity			Group comparison	Effect size in Hz
MPG-OOG	Source		Target		
*Inhibition*	RMFG	→	LSTG	+	0.06
	LMeFG	→	LIFG	+	0.09
	LMeFG	→	RMFG	+	0.13
	LMeFG	→	LMTG	+	0.08
	LSTG	→	LIFG	+	0.13
	RSMG	→	LIFG	+	0.14
	RSMG	→	RIFG	+	0.08
	LIFG	→	RMFG	−	−0.11
	LIFG	→	RSMG	−	−0.09
	RIFG	→	LMeFG	−	−0.07
	RMFG	→	LIFG	−	−0.09
	RMFG	→	LMTG	−	−0.08
*Excitation*	RIFG	→	LIFG	+	0.08
	RIFG	→	LMTG	+	0.13
	RMFG	→	RSMG	+	0.25
	LSTG	→	LMeFG	+	0.09
	LSTG	→	RSMG	+	0.17
	RSMG	→	RMFG	−	−0.18
	LIFG	→	LMTG	−	−0.09
	LIFG	→	LSTG	−	−0.18

*Note.* MPG = male perpetrator group; OOG = other offender group. Group comparison is always in relation to male perpetrators. Increased connectivity in MPG compared to OOG is represented by " + ", while decreased connectivity in MPG compared to OOG is represented by "-". Left inferior frontal gyrus (LIFG), right inferior frontal gyrus (RIFG), right middle frontal gyrus (RMFG), left medial frontal gyrus (LMeFG), left middle temporal gyrus (LMTG), left superior temporal gyrus (LSTG) and right supramarginal gyrus (RSMG)

#### Association between the ability to down-regulate emotions in response to IPVAW-related stimuli and effective connectivity in male perpetrators

After determining the neural differences between groups, we were specifically interested in how the effective connectivity at rest was related to the behavioural ability to down-regulate emotions in response to IPVAW-related stimuli within the male perpetrator group. The results of this analysis are shown in Fig. 4b and reported in Table 5.

**Table 5:** Significant associations between the ability to down-regulate emotions in response to IPVAW-related stimuli and effective connectivity in male perpetrators

Network	Connectivity			Association with covariate	Effect size in Hz
	Source		Target		
*Inhibition*	LIFG	→	RMFG	+	0.31
	LIFG	→	LSTG	+	0.10
	LIFG	→	RSMG	+	0.16
	RIFG	→	LMeFG	+	0.07
	RMFG	→	LSTG	+	0.18
	LMTG	→	RIFG	+	0.12
	LMTG	→	RMFG	+	0.10
	LMTG	→	LMeFG	+	0.11
	RMFG	→	LIFG	−	−0.24
	RMFG	→	LMeFG	−	−0.21
	LMTG	→	LSTG	−	−0.12
	LSTG	→	LIFG	−	−0.11
	RSMG	→	LIFG	−	−0.09
*Excitation*	LIFG	→	LMeFG	+	0.11
	RMFG	→	RSMG	+	0.35
	LMeFG	→	RMFG	+	0.09
	RSMG	→	RIFG	+	0.12
	LIFG	→	LMTG	−	−0.14
	RIFG	→	RSMG	−	−0.21
	LMeFG	→	LIFG	−	−0.21
	LMeFG	→	RIFG	−	−0.10
	LMeFG	→	LSTG	−	−0.21
	LSTG	→	RMFG	−	−0.12
	RSMG	→	LSTG	−	−0.07

MPG = male perpetrator group; association with the covariate: " + " represents a positive correlation between the specific effective connectivity and the covariate of interest, while "-" represents a negative correlation. Left inferior frontal gyrus (LIFG), right inferior frontal gyrus (RIFG), right middle frontal gyrus (RMFG), left medial frontal gyrus (LMeFG), left middle temporal gyrus (LMTG), left superior temporal gyrus (LSTG) and right supramarginal gyrus (RSMG)

Within the reappraisal network, half of the connections were positively associated with the ability to down-regulate emotions in response to IPVAW-related stimuli (12/24). Connections within frontal regions, specifically those coupling the LIFG to RMFG/LMeFG, were positively associated with the covariate of interest. In contrast, connections in the opposite direction (from RMFG/LMeFG to bilateral IFG) were negatively associated with the ability to down-regulate emotions in response to IPVAW-related stimuli. Regarding fronto-temporoparietal projections, connections from the LIFG/RMFG to LSTG/RSMG were positively associated with the covariate. In contrast, projections from the RIFG/LMeFG to LSTG/RSMG demonstrated a negative association with the ability to decrease the emotional state. Moreover, most of all connections from the temporoparietal to frontal regions were negatively associated with the ability to down-regulate emotions in response to IPVAW-related stimuli, except for those involving LMTG. Finally, connections within temporoparietal areas were also negatively associated with the covariate. Overall, 54% of the connections were inhibitory, and 46% were excitatory.

Additionally, from all connections demonstrating a link between effective connectivity and the ability to down-regulate emotions in the male perpetrator group, 13 connections also demonstrated a difference in connectivity strength in comparison to the other offender group. This means the same connections that showed a group difference in effective connectivity were additionally linked to the ability to down-regulate emotions in the male perpetrator group (illustrated in Fig. 4b by black squares). These connections included: all projections from frontal to temporoparietal regions except for the connection from the RIFG to RSMG and the LMeFG to LSTG, and mostly within prefrontal connections as well as connections from temporoparietal to frontal areas overlapped with the comparison between MPG and OOG. LIFG was the brain region with the most input and output connections in both models.

#### Prediction of the ability to down-regulate emotions in response to IPVAW-related stimuli from male perpetrators' effective connectivity

**Fig. 5:**
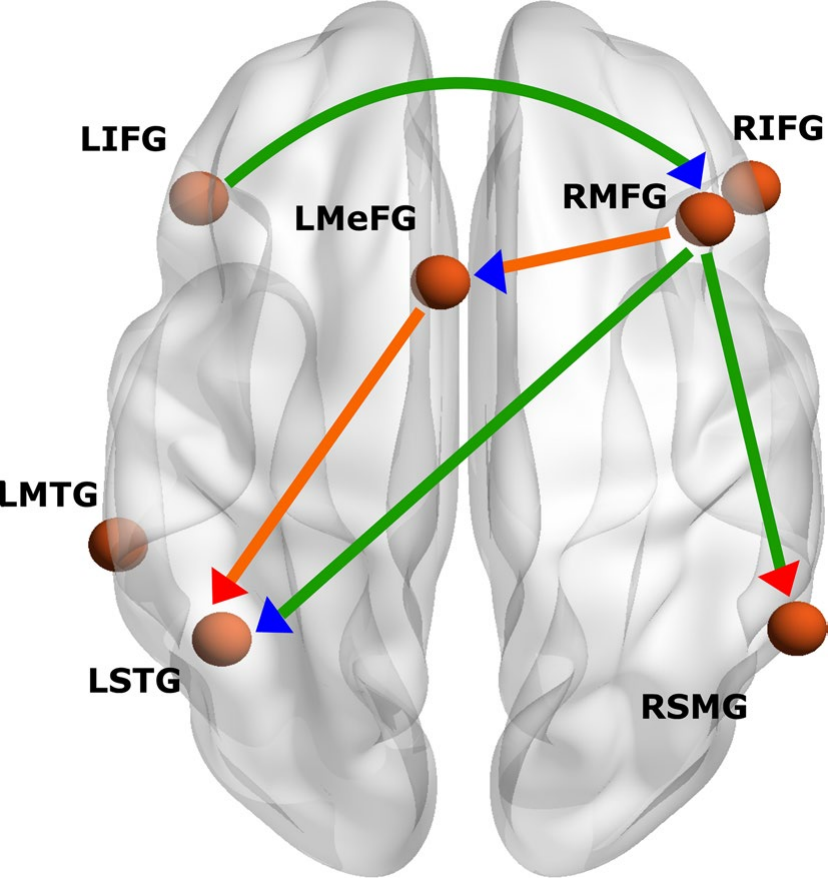
Leave-one-out cross-validation results. Only significant effect sizes (**p** < .05) to predict the ability to down-regulate emotions in response to IPVAW-related stimuli are represented. Green/orange arrows represent a positive/negative association between the ability to down-regulate emotions and effective connectivity in MPG. Red/blue triangles indicate excitatory/inhibitory connections. LIFG = left inferior frontal gyrus; RIFG = right inferior frontal gyrus; LMeFG = left medial frontal gyrus; RMFG = right middle frontal gyrus; LMTG = left middle temporal gyrus; LSTG = left superior temporal gyrus; RSMG = right supramarginal gyrus

Finally, we assessed whether the ability to down-regulate emotions in response to IPVAW-related stimuli could be predicted by the effective connectivity within the male perpetrator's group. A threshold of a posterior probability of > 0.95 was used to select the connections for the LOOCV. The analysis revealed that effect sizes were large enough to predict the ability to down-regulate emotions in response to IPVAW-related stimuli with an out-of-sample estimate for five connections: from the LMeFG to LSTG, from the LIFG to RMFG and, from the RMFG to LSTG, LMeFG and RSMG. In addition, all of these connections demonstrated a difference in connectivity between MPG and OOG (except for the RMFG to LMeFG and LMeFG to LSTG projections). Results are shown in Fig. 5 and reported in Table 6.

**Table 6:** Significant associations after cross-validation between the ability to down-regulate emotions in response to IPVAW-related stimuli and EC in male perpetrators

Network	Connectivity			Association with covariate	Pearson's r	*p-value*
	Source		Target			
*Inhibition*	LIFG	→	RMFG	+	.470	.010
	RMFG	→	LSTG	+	.379	.033
	RMFG	→	LMeFG	−	.356	.044
*Excitation*	RMFG	→	RSMG	+	.400	.026
	LMeFG	→	LSTG	−	.358	.043

Left inferior frontal gyrus (LIFG), right inferior frontal gyrus (RIFG), right middle frontal gyrus (RMFG), left medial frontal gyrus (LMeFG), left middle temporal gyrus (LMTG), left superior temporal gyrus (LSTG) and right supramarginal gyrus (RSMG)

## Discussion

Here we studied for the first time whether intrinsic neural dynamics supporting reappraisal are specific to male perpetrators of IPVAW and its association with the ability to down-regulate emotions in response to IPVAW-related stimuli. Our results showed that both criminal groups (male perpetrators and other offenders) shared a specific reciprocal mechanism reflected in increased effective connectivity within prefrontal regions and increased effective connectivity from prefrontal to temporoparietal regions but reduced connectivity in the opposite direction. In addition, male perpetrators show different effective connectivity compared to other offenders. They simultaneously exhibited an increased connectivity from the ventrolateral prefrontal cortex and a decreased connectivity from the dorsolateral prefrontal cortex to the same TPJ/STG regions. They also showed enhanced connectivity from SMA to frontal and temporal areas. Finally, cross-validation analysis revealed that connections from LVLPFC to RDLPFC and from RDLPFC to temporoparietal areas could be possible predictors of the ability to down-regulate emotions in response to IPVAW-related stimuli in male perpetrators. The present study is the first attempt to explore whether the neural dynamics at rest are related to manifestations of emotion regulation difficulties in IPVAW perpetration.

### Do male perpetrators exhibit different effective connectivity within the reappraisal-related brain network compared to non-offenders and other offenders?

Our first main finding revealed a large amount of overlap in effective connectivity within the reappraisal brain network between male perpetrators and other offenders when comparing with non-offenders. Both convicted groups showed increased inhibitory connectivity within prefrontal areas (LVLPFC to RDLPFC), increased inhibitory connectivity from the prefrontal (LVLPFC) to temporoparietal regions (TPJ/STG) and reduced inhibitory connectivity in the opposite direction compared to men with no criminal records. These results extend the current knowledge of brain functioning in criminal populations. According to the neuromoral theory (Raine 2019), the aforementioned areas are core components of the moral brain network. Specific structural or functional alterations within this network would contribute to the generation of moral thoughts, emotions and conducts that underlie different antisocial or criminal behaviour (Raine 2019; Raine and Yang 2006). From this point of view, the increased effective connectivity within prefrontal regions aligns with prior resting-state and task-based fMRI studies on psychopaths and high-risk prisoners (Leutgeb et al. 2016; Rodríguez-Contreras et al. 2015; Glenn et al. 2009). In this line, the heightened intra-frontal connectivity at rest might reflect a compensatory mechanism that facilitates the enhanced activity of prefrontal regions during moral decision-making (Korponay et al. 2017; Yang et al. 2012). Furthermore, both convicted groups also showed increased effective connectivity from the prefrontal to parietal regions and reduced connectivity in the opposite direction. This finding suggests a specific bidirectional mechanism between top-down regulatory regions such as the LVLPFC (Morawetz et al. 2016) and TPJ/STG regions involved in the attribution of intentions (Decety and Lamm 2007). Considering the present results, both convicted groups share specific reappraisal-related neural dynamics that might be also involved in moral processing. In fact, a large body of literature has demonstrated that emotion regulation and morality are interdependent constructs (Zhang et al. 2017; Li et al. 2017; Szekely and Miu 2015) that share a common neural basis (Helion and Ochsner 2018; Harenski and Hamann 2006).

Although the two criminal groups seem to share similar neural network dynamics compared to non-offenders, they also differ in several aspects. It should be noted that variability within the group of other offenders (integrated by men convicted for robbery, drug trafficking and dangerous driving) might had some impact at a brain connectivity level, which may explain why male perpetrators demonstrate more differences when compared to other offenders than to non-offenders. Yet, a general reversed pattern regarding fronto-temporoparietal connections needs to be highlighted. We observed that male perpetrators exhibited decreased connectivity from LVLPFC but increased connectivity from RDLPFC to temporoparietal regions (TPJ/STG), being excitatory and inhibitory connections, respectively. These results are in line with our previous findings, demonstrating differences in functional connectivity and brain activation between male perpetrators and other offenders in these specific brain regions (Marín-Morales et al. 2022a; Amaoui et al. 2022). Importantly, male perpetrators also showed increased inhibitory connectivity from SMA to prefrontal and temporal regions compared to other offenders. SMA is involved in the creation of mental representations (Kohn et al. 2014), which makes it a core component of empathy and moral evaluation (Fan et al. 2011; Yoder et al. 2015). The increased effective connectivity between SMA–prefrontal regions could suggest that male perpetrators demonstrate a different intrinsic neural pattern supporting the reformulation and reconceptualisation of mental representations (Silvers and Guassi Moreira 2019). In line with this view, previous MRI studies showed that male perpetrators exhibited an over-activation of the SMA when processing general violence images compared to other criminals (Bueso-Izquierdo et al. 2016a) which was interpreted as a hyper-response to threatening situations (Lee et al. 2009). Taken together, dysfunctions in the neural dynamics underlying social representations could lead to an altered hyper-response to menacing situations.

### Does the ability to down-regulate emotions in response to IPVAW-related stimuli is associated with the intrinsic neural dynamics in male perpetrators?

This is the first study that uses a stimulus specifically related to the committed crime in an rs-fMRI study with male perpetrators. The advantage of considering this behavioural variable lies in the possibility to determine those neural connections that are particularly related to IPVAW offence. Although no clear pattern was found, nearly all connections in the network were modulated by the ability to down-regulate emotions in response to IPVAW-related stimuli, which might indicate that this ability is strongly linked to the underlying network architecture. Taking a step further, cross-validation analysis (Friston et al. 2014) revealed which connections might be potential predictors of the ability to down-regulate emotions in response to IPVAW-related stimuli. Special attention should be paid to RDLPFC, since we found it to be the most connected brain region, receiving inputs from LVLPFC and sending outputs to SMA, STG and TPJ (almost all connections were inhibitory). Considering the results, it seems that RDLPFC is a potential hub in the neural network supporting the ability to down-regulate emotions in response to IPVAW-related stimuli. It is well known that DLPFC manages higher-order control functions including monitoring and manipulating representations in working memory (Morawetz et al. 2016) which makes it a key region responsible for top-down regulation (Kohn et al. 2014) and control inhibition (Ochsner et al. 2012).

Furthermore, all connections involving the RDLPFC (except for the RDLPFC-SMA connection) positively predicted the ability to down-regulate the emotional states in response to IPVAW-related stimuli. In other words, the higher the effective connectivity within the male perpetrator group, the harder their ability to down-regulate emotions when facing IPVAW-related stimuli. This finding supports the hypothesis of an enhanced brain network associated with emotion regulation difficulties (Repple et al. 2017; Beyer et al. 2014).

### Limitations

The present study has some limitations to be acknowledged. First, the heterogeneity within the group of other offenders could be a source of variability in brain connectivity. In order to control this issue, both groups were equally matched in the severity of the committed crime. Future studies need to address whether men convicted for IPVAW differ specifically from men sentenced for violent crimes other than IPVAW. Additionally, taking the presence of drug dependence or brain damage as an exclusion criterion could be a limitation as it reduces the representativeness of the target group (Marín-Morales et al. 2021; 2022a). However, we believe this is the best way to study those specific factors that make IPVAW different from other types of violence. Our population of interest is not those men who have committed violence due to head injury or substance abuse, but those who have committed intimate partner violence due to the social factors that surround this reality (e.g., patriarchal system, sexism…). In addition, male perpetrators might exhibit high social desirability, which could explain the lack of differences between groups in the self-reports. Although the criminal groups were matched in terms of severity and duration of the sentence, the time spent in the Social Integration Centre (CSI) at the time of the assessment could not be controlled. This limitation opens avenues for future studies to investigate the impact of the duration of CSI involvement on the brain connectivity–behaviour relationship in these populations. These issues would need to be addressed in future studies.

## Conclusions

Our study provides the first evidence for a specific intrinsic neural network supporting reappraisal in men convicted of an IPVAW crime. The results corroborate that male perpetrators exhibit a different brain pattern related to reappraisal compared to non-offenders and other offenders (Marín-Morales et al. 2021). Our first key finding showed that male perpetrators and other offenders shared effective connectivity differences in comparison to non-offenders. This involved a specific bidirectional effect between prefrontal top-down regulatory core regions and temporoparietal areas that are responsible for the generation of social representations. While in the male perpetrators versus other offenders contrast, we observe a general inverted prefrontal to temporoparietal pattern, where we highlight the increased SMA to prefrontal effective connectivity in male perpetrators. The second key finding is that we identified possible predictors of the ability to down-regulate emotions in male perpetrators, although there is still a lot of variability to be explained. Specifically, connections involving the RDLPFC, by integrating inputs from LVLPFC and sending outputs to temporoparietal regions, seem to predict the ability to down-regulate emotions in response to IPVAW-related stimuli in male perpetrators, which reinforces the relevance of cognitive processing in emotion regulation (Golkar et al. 2012). According to the process model of emotion regulation (Gross 2015), the findings suggest that men convicted of IPVAW present differences in connectivity between regions that serve the ability to identify an alternative interpretation of the emotional-inducing cue when it comes to IPVAW situations. This strategy allows one to change the course of the emotional response, thus reducing the probability of using violence as a conflict-solution strategy. We want to emphasise the importance of paying attention to differences in emotion regulation as a risk factor in IPVAW perpetration and therefore encourage future research to explore other regulatory strategies and their neural underpinning taking into consideration subgroups of perpetrators based on the risk of reoffending. This knowledge could inform the work of adaptive emotion regulation strategies in intervention programmes for male perpetrators in order to reduce the high recidivism rates to fight against IPVAW.

### Supplementary Information

Below is the link to the electronic supplementary material.

Supplementary file1 (DOCX 525 KB)

## Data Availability

The datasets generated during and/or analysed during the current study are not publicly available but are available from the corresponding author at reasonable request.

## Footnotes

1 An amendment to the preregistration was made after analysing the results of the emotion regulation fMRI task (Marín-Morales et al. 2021). The preregistered variable was modified as it did not reflect the specificity of the population nor the emotion regulation strategy of interest. The new behavioural variable is explained in the Analysis section.
