# Supplementary material for: Intrinsic neural network dynamics underlying the ability to down-regulate emotions in male perpetrators of intimate partner violence against women
Source: Brain Struct Funct. 2023 Sep 9;228(9):2025–40. doi: 10.1007/s00429-023-02696-x (PMC10587320; doi:10.1007/s00429-023-02696-x)
Supplement: Supplementary file 1 — Supplementary file1 (DOCX 525 KB) [file 429_2023_2696_MOESM1_ESM.docx]

**SUPPLEMENTAL FILES**

**Intrinsic neural network dynamics underlying the ability to down-regulate emotions in male perpetrators of intimate partner violence against women**

**Emotion regulation task**

The emotion regulation task described in detail in (Marín-Morales et al. 2021) was composed of forty stimuli: 8 neutral pictures, 16 negative pictures unrelated to IPVAW (e.g., injured animals, men in a war), and 16 IPVAW-related stimuli (e.g., men threatening a woman, men hitting their female partner). Three conditions were implemented in the task:

1. In the ‘Observe’ condition, participants had to passively observe neutral pictures, without trying to manipulate their own emotional state.
2. In the ‘Increase’ or ‘Experience’ condition, participants were asked to increase their emotional state's intensity by reappraisal in response to IPVAW-unrelated pictures or IPVAW-related pictures. Participants were asked to up-regulate the emotional impact by putting themselves in the actors’ position. For example: After the instruction “Experience,” and during the viewing of a picture of a man intubated at the hospital: “Imagine that you are that person and you are living that painful situation, think that you are the one in the picture. Feel and be aware of your emotions.”
3. In the ‘Decrease’ or ‘Suppress’ condition, participants were asked to reduce the intensity of their emotional state by using reappraisal in response to IPVAW-unrelated pictures or IPVAW-related pictures. They were instructed to down-regulate the emotional impact by taking an external perspective, distancing from the experience or thinking that the situation is not real. For example, after the instruction “Suppress” and during the visualization of a picture of a disfigured man the participant was told: “At the very moment the picture appears, try to transform the negative emotion generated by that picture by reinterpreting it: imagine he is an actor wearing make-up.”

All participants had pre-scanner training, where they practised reappraisal strategies. After the regulation phase, participants had to rate the intensity of the experienced emotion on a scale ranging from 1 (slightly unpleasant) to 5 (very unpleasant).

**Resting-state functional MRI data acquisition**

All participants underwent MRI scanning at the Mind, Brain and Behavior Research Center (Granada, Spain). Resting-state images were acquired using a 3-T Siemens Trio MRI scanner with a 32-channel whole brain coil. First, high-resolution T1-weighted anatomic images were acquired using a three-dimensional weighted turbo-gradient-echo sequence in the sagittal orientation. Acquisition parameters were: Repetition time (TR) = 2300 ms; Echo time (TE) = 3.1 ms, Field of view (FOV) = 208; Voxel size = 0.8 x 0.8 x 0.8 mm, 208 slices. Functional images were acquired using a T2*-weighted echo-planar imaging (EPI) sequence. A total of 240 whole-brain volumes were recorded with the following parameters: TR = 2.0s; TE = 25 ms; FOV = 238 x 238 mm2; Acquisition Matrix = 68 x 68; thirty-five 3.5m axial slices, Voxel Size = 3.5 x 3.5 x 3.5 mm3. All participants were asked to close their eyes and try not to think of anything particular during the 8 minutes scan.

**Sample size for emotion regulation self-reports:**

| **Variables** | | **MPG** | **OOG** | **NOG** |
| --- | --- | --- | --- | --- |
| **DERS** | | | | |
|  | Non acceptance of negative emotional responses | 26 | 28 | 28 |
|  | Difficulties engaging in goal-directed behaviour | 25 | 29 | 29 |
|  | Difficulties controlling impulsive behaviour | 26 | 29 | 27 |
|  | Lack of emotional awareness | 24 | 28 | 29 |
|  | Limited access to ER strategies | 24 | 28 | 25 |
|  | Lack of emotional clarity | 25 | 27 | 28 |
| **ERQ** | | | | |
|  | Cognitive reappraisal | 24 | 29 | 29 |
|  | Expressive suppression | 25 | 28 | 29 |

*Note.* MPG = male perpetrator group; OOG = other offender group; NOG = non-offender group DERS = Difficulties in Emotion Regulation Scale; ERQ = Emotion Regulation Questionnaire.

**Supplemental Figure S1.** Posterior correlations among all parameters estimated from spectral DCM.
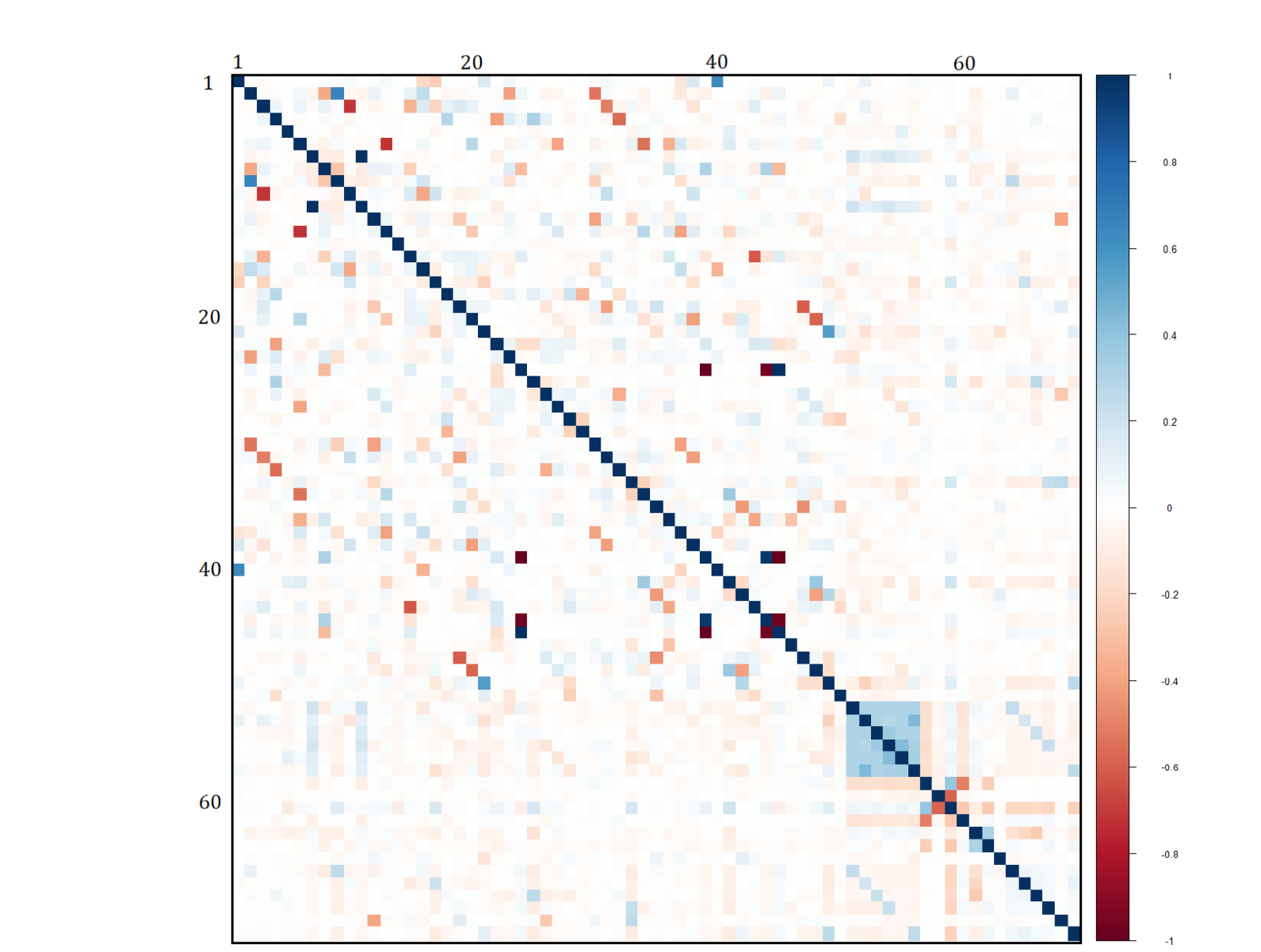


**Supplemental Figure S2.** First-level DCM model convergence statistics. **A.** Predicted variance explained for each participant. **B.** the largest absolute parameter estimate. **C**. The effective number of parameters in terms of divergence between the posterior and prior densities over the parameters.

**A. B.**
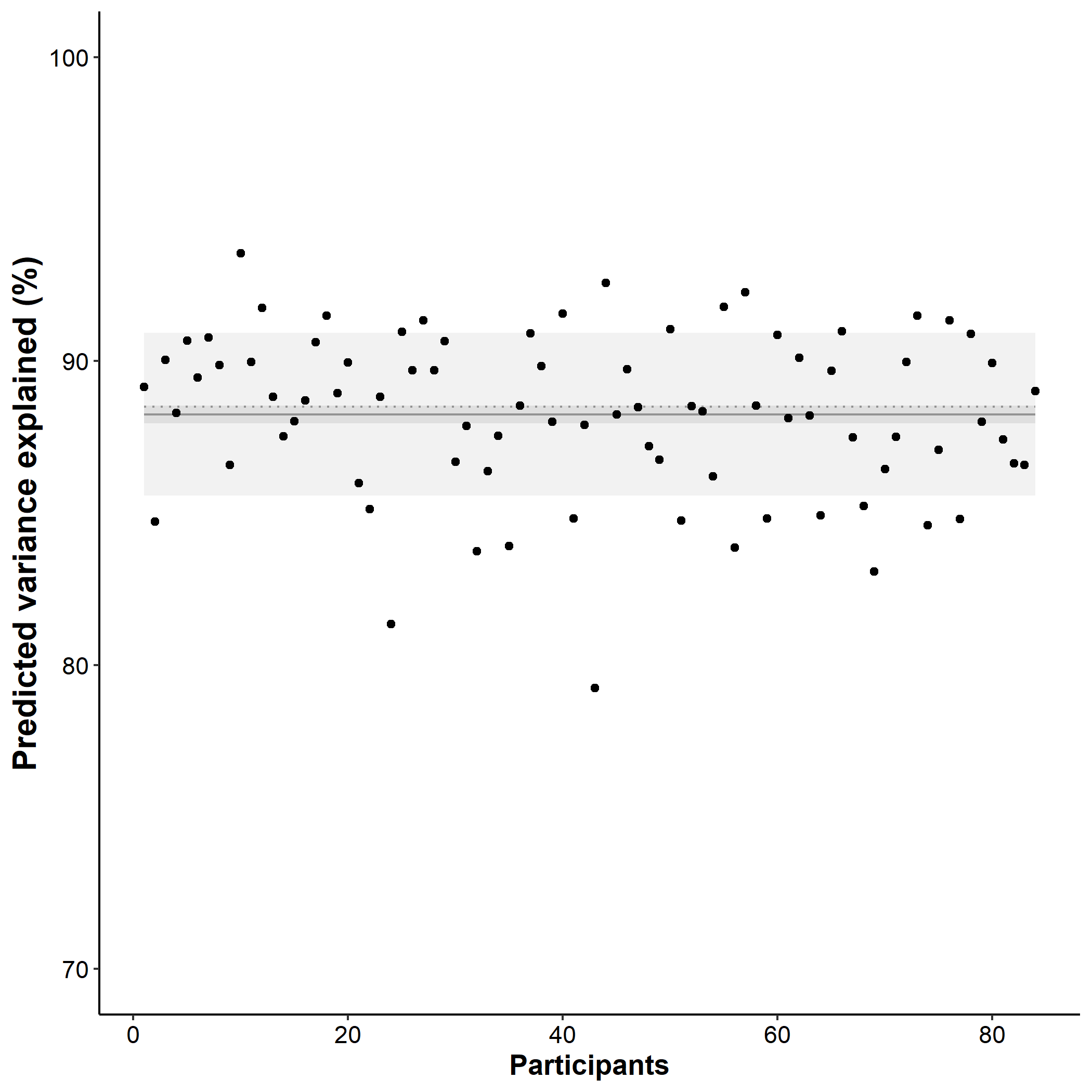


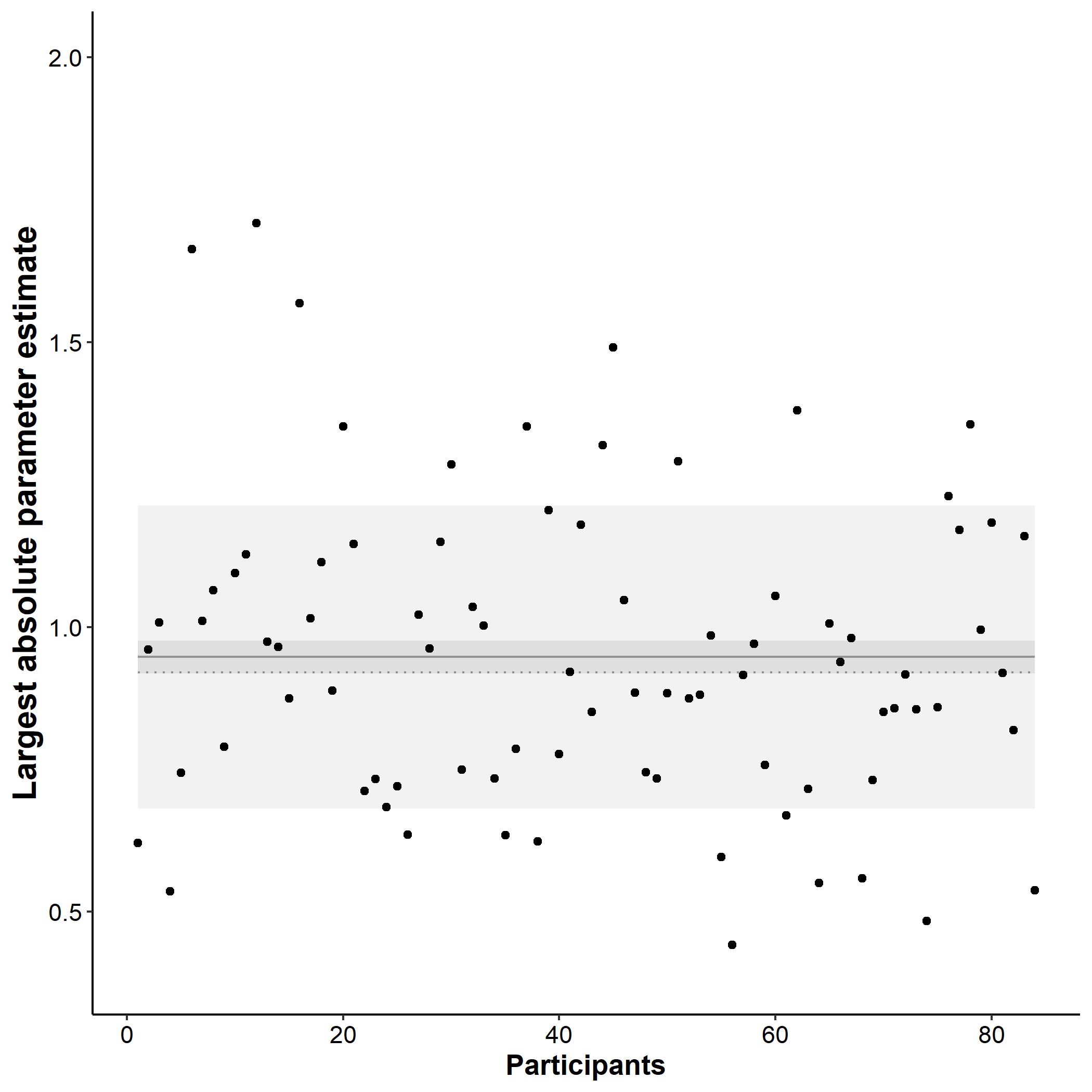


**C.**
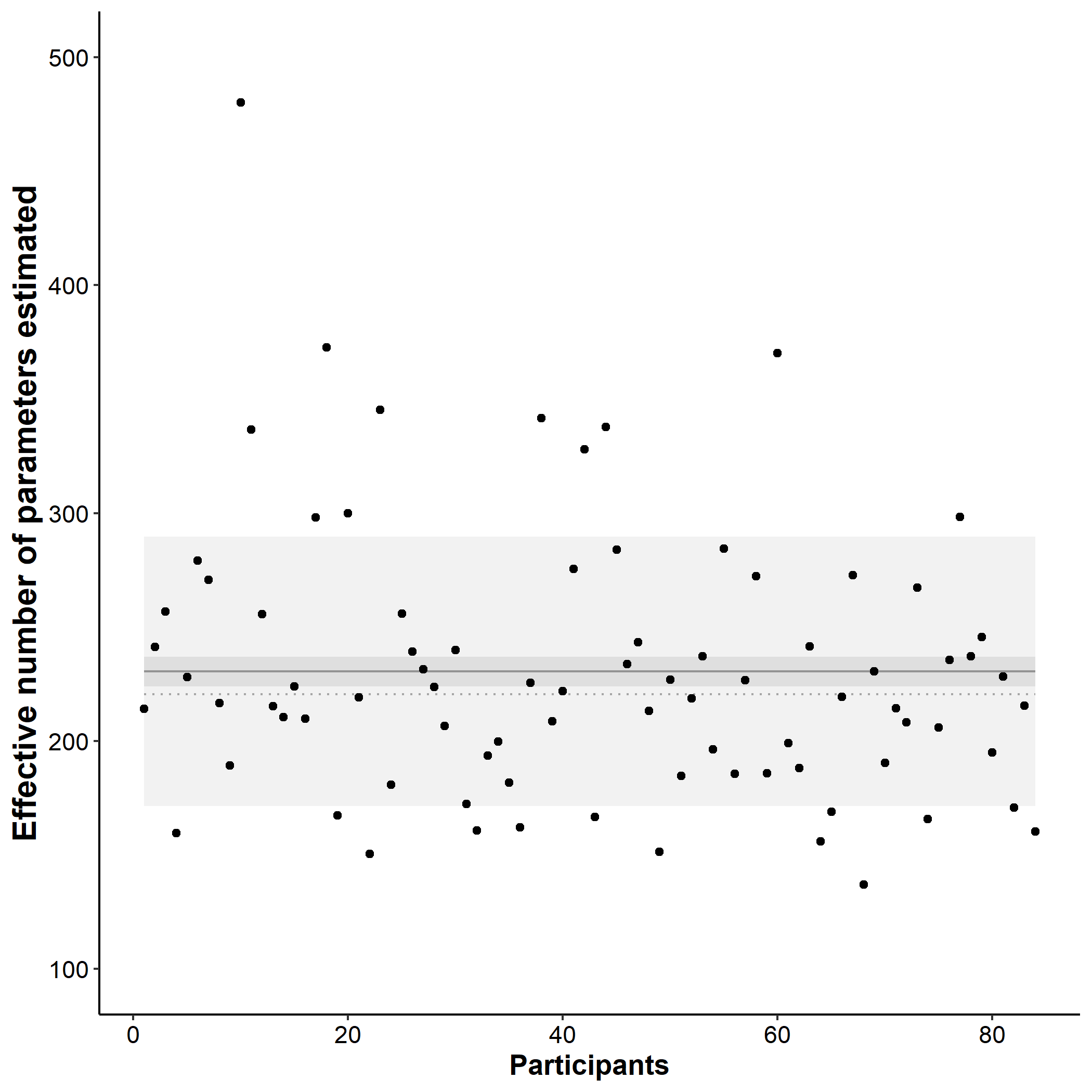


**Supplemental Figure S3.** Chord diagrams for group comparisons.


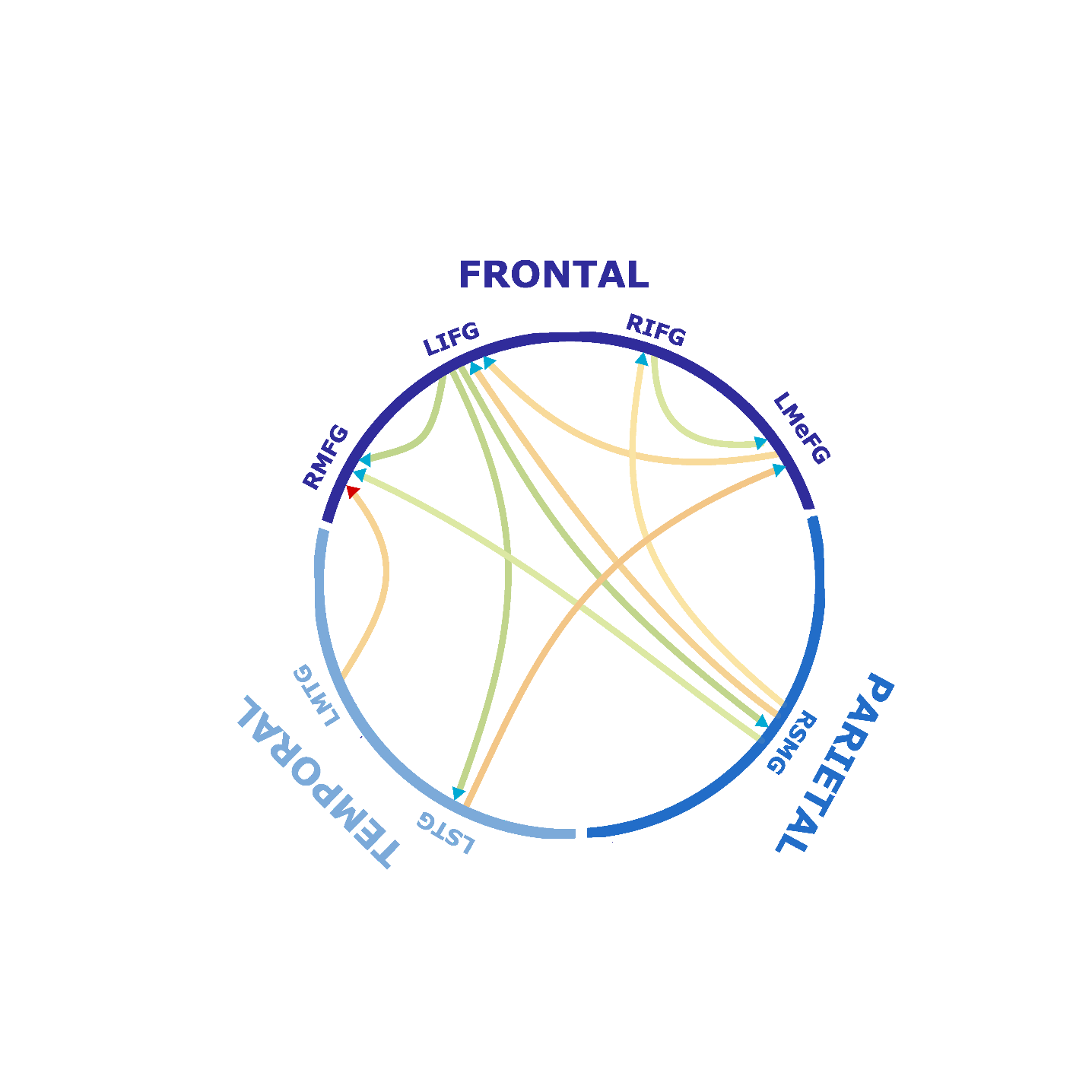


**A.**


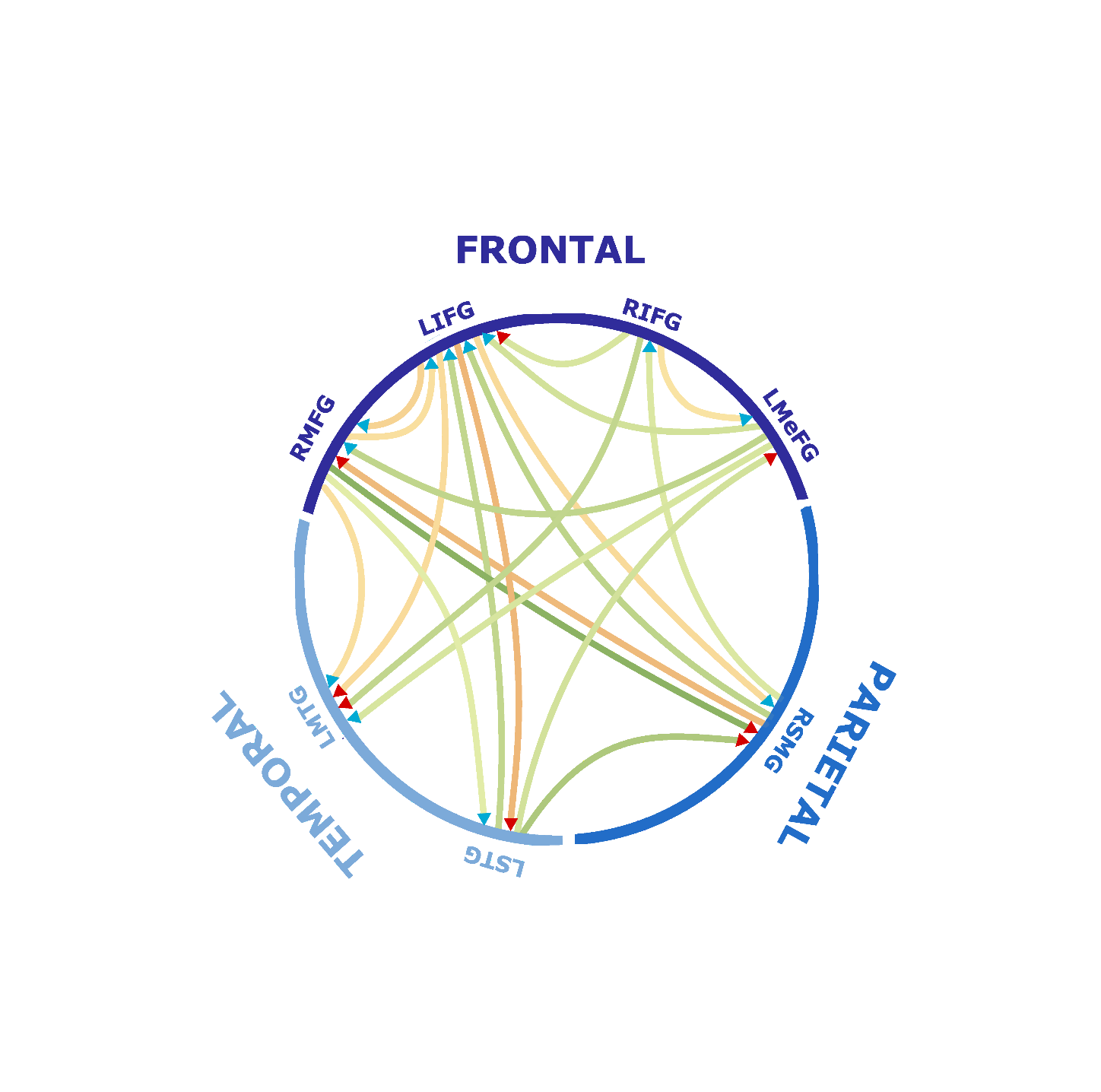


**B.**

**C.**
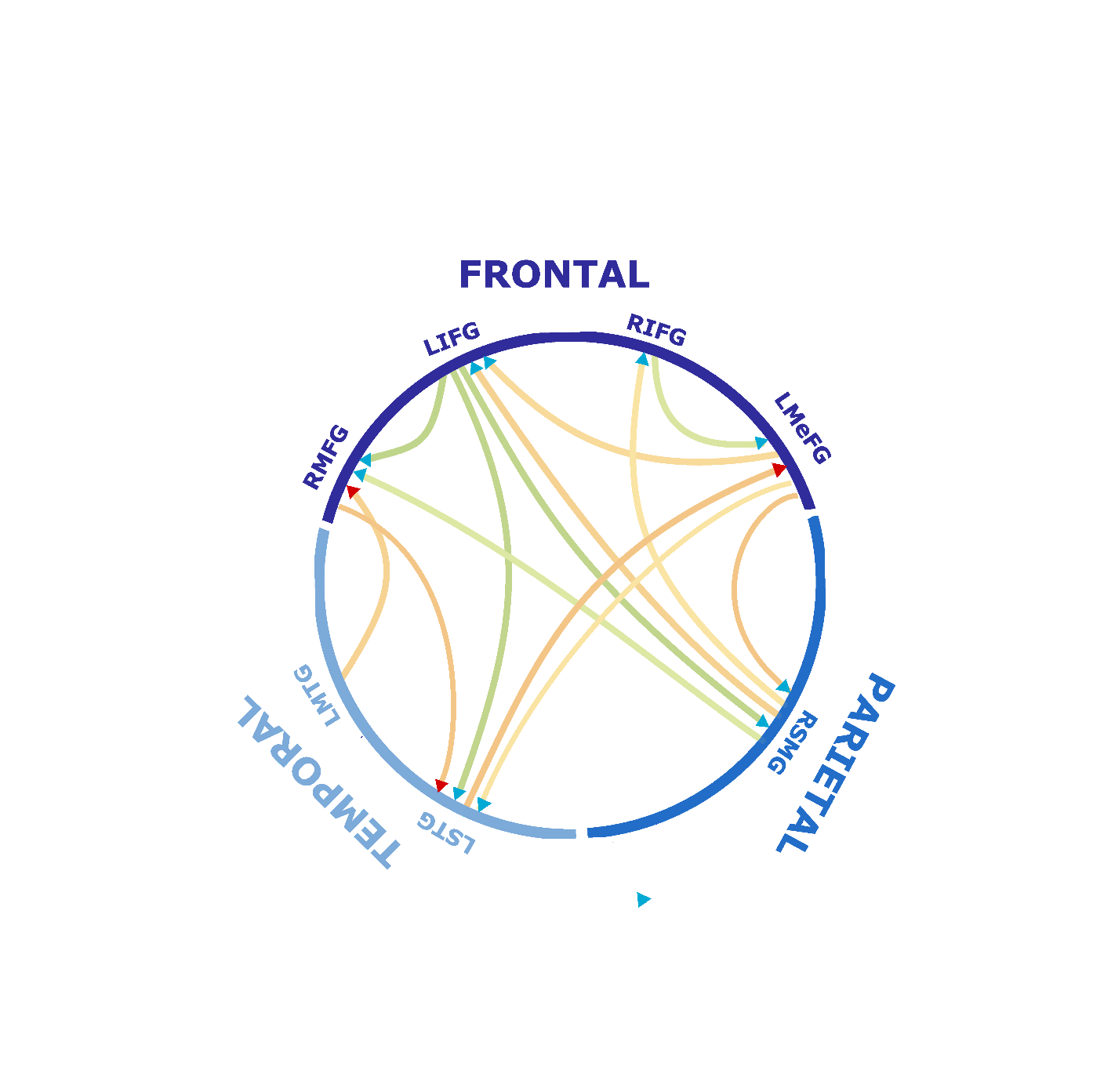


**A.** Differences in effective connectivity between MPG and NOG. **B.** Differences in effective connectivity between MPG and OOG. **C.** Differences in effective connectivity between OOG and NOG. Green: increased connectivity of MPG or OOG compared to control groups. Orange: decreased connectivity of MPG or OOG compared to control groups. Red triangle represents inhibitory connectivity and the blue triangle represents excitatory connectivity. LIFG = Left inferior frontal gyrus; RIFG = Right inferior frontal gyrus; LMeFG = Left medial frontal gyrus; RMFG = Right middle frontal gyrus; LMTG = Left middle temporal gyrus; LSTG = Left superior temporal gyrus; RSMG = Right supramarginal gyrus.
